# Dr. Sugrue, on Asthma

**Published:** 1800-10

**Authors:** C. Sugrue

**Affiliations:** Cork


### *To* Dr. BRADLEY.

SIR,

IN one of the Numbers of your Journal, Dr. Stoll's practice of suspending the progress of phthisis, by giving antimonials in nauseating doses, is mentioned; and it appears to be inferred from the success attending this practice, that the different preparations of this semi-metal act in a manner analogous to digitalis. As the attention of the faculty to this powerful medicine has been much increased by your valuable Publication, I have had several opportunities of observing its effects; and I believe myself authorised to conclude, that the *modus operandi* of the digitalis purpurea, is very different from that of the other medicines employed in the cure of pulmonary affections.

Asthma is a disease in which I was particularly induced to administer it, on account of the precarious effects of the medicines usually given. In every case in which it was exhibited, the most violent symptoms were mitigated, and the general neral ſtate of health viſibly improved. One effect, however, particularly attracted my attention, which ſhewed how different its action was from that of antimonials, and which took place in every patient, viz. *The expectoration was diminiſhed, and at the ſame time the neceſſity of it ſeemed to be removed.* This effect was ſo remarkable, that ſome of theſe aſthmatic patients, who had at different times received temporary benefit from garlic, ipecacuanha, and other medicines which increaſed expectoration, were ſurpriſed at finding relief from a medicine, the effects of which were ſo oppoſite. It appeared leſs efficacious in relieving the ſymptoms of aſthma, in thoſe caſes in which it produced nauſea or vertigo ; another ſtriking difference between its action and that of antimonials.

The increaſe of pulmonary abſorption by this medicine, to which Dr. Darwin aſcribes its ſalutary effects, is certainly a more ſatisfactory explanation of its *modus operandi* than any other that has been attempted. But were we to adopt his opinion, that the increaſed action of the pulmonajy abſorbents was the effect of the decreaſed action of the ſtomach, we ſhould infer, that this medicine would be more efficacious in proportion to the degree of nauſea, or even of vomiting, produced by it; the contrary of which is, however, the fact.

Not having met with a circumſtantial narrative of any caſe of aſthma, in which digitalis was given, as we are not able to procure the medical publications of your country until a conſiderable time has elapſed, I ſhall beg leave to ſubjoin a ſhort account of a caſe, in which its ſalutary effects were ſpeedily and deciſively produced.

Daniel Sheehan, æt. 56, a gardener, had contracted an aſthma about twelve years ago, in conſequence of expoſure to alternations of heat and cold while working in a cellar in this city. The ſenſe of ſuffocation on aſcending an eminence, and the oppreſſion and ſqueezing of the cheſt he felt during the greater part of the night, gradually but ſlowly increaſed until Chriſtmas laſt; at which time having caught freſh cold, theſe ſymptoms were very much aggravated. He called on me in the beginning of May laſt, very pale and emaciated; he complained very much of the ſenſe of ſuffocation, and tightneſs about the cheſt; he ſcarcely ſlept; but after dozing about an hour on going to bed, he awoke very much oppreſſed, was obliged to ſit up in bed during the remainder of the night, and very often believed that he could not live until morning. His pulſe was about 120, and very feeble.

I ordered him fifteen drops of Darwin's tincture of digitalis twice a day. As he lives in a remote part of the city, I did not ſee him for a fortnight; when he called upon me, I was ſtruck

ſtruck by the remarkable change in his appearance; he had no longer the wheezing nor oppreſſion about his cheſt; his countenance was much fuller, and his complexion much leſs pale; his pulſe was about 90, and tolerably ſtrong. He told me, that after he had taken the bottle about three days, he no longer felt himſelf obliged to ſit up at night, but was able to take a comfortable nap, after which he felt himſelf refreſhed, a ſenſation with which he had been for ſome months unacquainted. At the expiration of a week, he could ſleep five or ſix hours, and his appetite and ſtrength improved in the ſame proportion; he no longer experiences the neceſſity of ſtopping to take breath on aſcending an eminence. He ſtill continues the medicine; and in his own opinion, and that of his family, his general health is better than it had been at any period during theſe ten years. I remain,

Your's, &c.

*Cork, June* 18, 1800. C. SUGRUE, M.D.

